# Breast cancer burden in eastern Sudan: seven-year retrospective study

**DOI:** 10.3332/ecancer.2024.1704

**Published:** 2024-05-21

**Authors:** Ahmed Balla M Ahmed, Salma Alrawa, Ahmed A Yeddi, Esraa S A Alfadul, Hind Mohi Aldin Abd Allah, Muhannad Bushra Masaad Ahmed³

**Affiliations:** 1 Faculty of Medicine, University of Khartoum, Khartoum 11111, Sudan; 2 East Oncology Center, Ministry of Health, Gadarif 11111, Sudan; 3 Faculty of Medicine and Health Sciences, University of Gadarif, Gadarif 11111, Sudan

**Keywords:** breast cancer, burden, oestrogen receptor, Sudan

## Abstract

**Background:**

Breast cancer (BC) is prevalent in Sudan, yet data on its epidemiology in Eastern Sudan is limited. This study aims to provide insights into the demographic and clinicopathologic features of BC patients treated at the East Oncology Centre (EOC) in Gadarif State, Eastern Sudan. Furthermore, we aim to identify the factors that contribute to a late-stage diagnosis.

**Methods:**

This cross-sectional study included patients diagnosed with BC and treated in the EOC between 2016 and 2022. Data obtained from medical records were analysed using R software, with descriptive statistics and multiple logistic regressions applied to determine determinants of advanced-stage presentation. A *p*‐value < 0.05 was considered statistically significant.

**Results:**

Among the 394 patients studied, the majority were women (96%), married (66%) and from rural areas (43%). The peak years for BC diagnoses were 2018 and 2022, with a median age at diagnosis of 48 years. A family history of cancer was reported by 20% of patients. Clinical stages were distributed as follows: I (1.6%), II (17%), III (50%) and IV (32%). Twenty-five percent tested positive for human epidermal growth factor receptor 2, while 73% tested negative and 43% had triple-negative BC. Modified radical mastectomy was performed in 47% of patients, with 21% undergoing breast-conserving surgery. Treatment rates were 38% for radiotherapy, 84% for chemotherapy and 46% for hormonal therapy. Higher grade BC and lower education levels were associated with advanced-stage presentation, while a family history of cancer reduced the risk of advanced-stage disease (OR: 0.38, 95% CI: 0.18–0.78).

**Conclusion:**

The study found that females in East Sudan often present at a young age and advanced stage, with a significant prevalence of triple-negative BC. Notably, family cancer history exhibited a protective effect against advanced-stage presentation, while grade 3 cancer was positively associated with advanced disease.

## Introduction

Globally, breast cancer (BC) emerged as the most frequently diagnosed malignancy in women, with an estimated 2.3 million new cases (11.7% of total cases) in the year 2020 [1].

BC is a complex disease with diverse clinical and pathological characteristics, showing significant variations in its presentation, histologic type, molecular biomarkers, prognosis and treatment outcomes [2]. The stage at which the disease is diagnosed strongly influences overall survival and mortality rates. Approximately 54% of women are diagnosed in stage II, while only 16% are diagnosed in stage I [3].

In Africa, BC accounts for 186,598 new cases, comprising approximately 17% of the total cancer cases in the region based on GLOBOCAN 2020 estimates [4]. Although it is less prevalent in Africa compared to Western industrialised nations, the mortality rates are alarmingly high [5]. The stage at which the disease is diagnosed strongly influences survival and mortality rates [3]. There is a concern that most African women with BC present at later stages and lack hormonal receptor expression [6].

Cancer estimates in Sudan rely mainly on data from hospital case series since there is no official population-based cancer registry in the country [6, 7]. Studies have shown that BC is the most common cancer among Sudanese women [8, 9], similar to other sub-Saharan African nations. These studies have also found that Sudanese women, like their counterparts in developed countries, tend to be diagnosed with BC at a younger age, at advanced stages, and with higher tumour grades [3, 7, 10]. A recent study from central Sudan revealed that patients' education level and duration from identification of BC symptoms to diagnosis significantly impact the stage at presentation time [10].

Little information is available about BC in the Gadarif Estate, Eastern Sudan. Therefore, the present study aimed to provide baseline information about the demographic features and tumour characteristics and investigate the associations between demographic variables and presentation stage in BC patients attending the East Oncology Centre (EOC), the main cancer treatment centre in this region.

## Methods

This is a facility-based cross-sectional study implemented using medical records of patients with BC treated at the EOC between 2016 and 2022. Cases were required to have histologically confirmed BC for inclusion in the study. Reviewing patients' medical files retrieved demographic, clinical and pathologic data.

### Study setting

The EOC was established in 2016 and is located in Gadarif City, the capital of Gadarif State, Eastern Sudan. The cancer treatment modalities available at the EOC include chemotherapy, hormonal therapy and palliative care. All patients with BC are reviewed at weekly BC multidisciplinary team meetings at the EOC in collaboration with the surgery department at Gadarif Teaching Hospital.

### Data collection method

All patients with BC who had been treated at the EOC during the study period were identified. A structured form was used to collect data from patients' medical files. These data included sociodemographic data (e.g., age, gender, education status, residence, family history of cancer, height, weight, marital status, age at menarche, age at menopause, age at first birth and parity); clinical characteristics (presenting complaints, stage at diagnosis according to the American Joint Committee on Cancer 2009 system and background medical history); pathologic features (histopathologic subtype and grade); molecular biomarkers (expression of oestrogen receptor (ER), progesterone receptor (PR) and human epidermal growth factor receptor 2 (HER2) status) and types of treatment.

### Ethical consideration

This study’s ethical approval was obtained from the ethics committee, Ministry of Health, Gadarif State, on 1 September 2023, under approval number RC.Q3.1.9.23. All files were anonymized and assigned an ID number.

### Data management and analysis

The data were transferred into an Excel spreadsheet and then imported into R software version 4.2.2. The normality of the data was checked by examining histograms. Descriptive statistics were used to analyse the frequencies and percentages for categorical variables, as well as the median and interquartile range for continuous variables. The study focused on assessing the burden of BC at EOC and examining the BC-related characteristics of female patients. Multiple logistic regression was utilised to identify the factors associated with advanced stage BC. The goodness-of-fit of the logistic model was evaluated using Hosmer and Lemeshow's test. A significance level of < 0.05 was considered for this research.

## Results

### Sociodemographic characteristics

**Figure 1.**
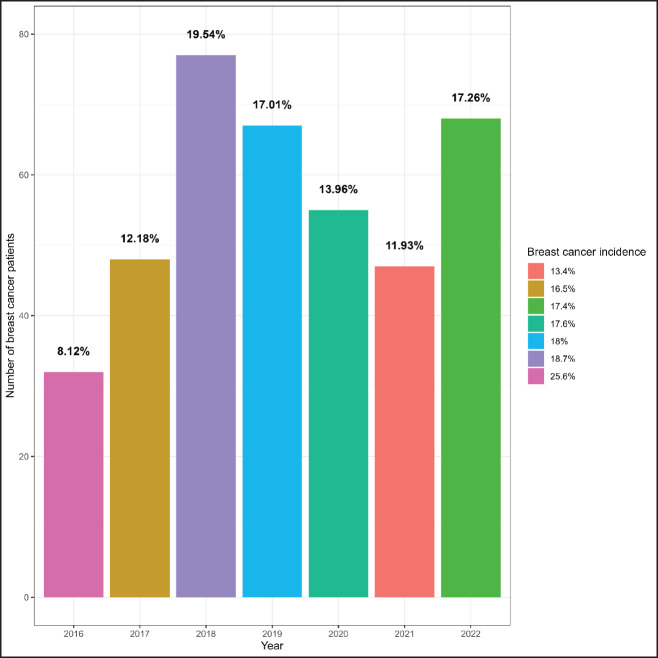
Burden of breast cancer at East Oncology Center from 2016–2022.

The study included 394 BC patients, primarily females (96%), housewives (81%), married (66%), illiterate (30%) and residing in rural Gadarif (43%) Table 1. In 2018, there were 77 cases of BC admissions, accounting for 19.5% of the patient cohort. There was a subsequent resurgence in cases in 2022, following a decline. The highest incidences of BC compared to other malignancies were observed in 2016 (25.6%) and 2018 (18.7%) Figure 1. Among female patients, the median age at menarche, menopause, childbirth and diagnosis was 14, 45, 20 and 48 years, respectively. The parity distribution showed that 42% had 1–3 children, 39% had 4–7 children and 19% had 8 or more children. Additionally, 20% had a family history of cancer Table 2.

### Clinical and pathological characteristics of BC and treatment modalities of the participants

The distribution of body mass index (BMI) showed that 12% were classified as underweight, 45% had a healthy weight, 29% were overweight and 14% were obese. There were 121 cases with missing BMI data. BC staging revealed that 1.6% were in stage I, 17% in stage II, 50% in stage III and 32% in stage IV. The tumour grade distribution showed that 8.1% were in grade one, 46% were in grade two and 46% were in grade three. Twenty-five percent were Her2 positive, while 73% were Her2 negative and 43% had triple-negative BC. Surgery types included: 47% who underwent modified radical mastectomy and 21% opted for breast conservative surgery. In terms of treatment, 38% received radiotherapy, 84% underwent chemotherapy and 46% received hormonal therapy. Palliative treatment was administered to 40% of participants Table 2.

### The associations between demographic variables and clinical stage

The study found that grade three BC significantly increased the risk of advanced stage compared to grade one (OR: 3.78, 95% CI: 1.30–10.9). Secondary school (OR: 0.28, 95% CI: 0.10–0.70) reduced the risk of advanced stage compared to illiteracy. Additionally, a family history of cancer (OR: 0.38, 95% CI: 0.18–0.78) lowered the risk of advanced-stage BC Table 3.

## Discussion

Sudan had only two healthcare facilities that provided cancer care until 2008, when the government started to establish other cancer centres [11]. Between 2008 and 2021, 12 cancer centres were established; among them, 2 were in Eastern Sudan, and both provided medical oncology services only. They were established in 2015 and started to function in 2016 [11]. The aim of this study was to assess sociodemographic characteristics, clinical characteristics and utilisation of cancer services in the EOC in Sudan.

Between 2016 and 2022, the EOC served 394 BC patients. The centre started with 32 patients in 2016, and this increased to 77 patients in 2018. The number of newly diagnosed patients was the lowest in 2020 and 2021. This can be attributed to the COVID-19 infection, which discouraged patients from seeking immediate medical care, as noted in other African countries [12]. It is worth noting that 4.3% of BC cases occur in males. This is higher than the 1% commonly reported in the literature [13–15]. It is also higher than the 2.2% reported in central Sudan [10]. The cause of this higher percentage should be studied.

The median age of diagnosing BC in Eastern Sudan is less than 50 years, similar to what has been reported from previous studies in Sudan [7], Arab countries [16] and Sub-Saharan Africa [17]. In the UK and USA, the majority of BC patients are diagnosed after 50 years [18, 19]. The difference in age at diagnosis is explained by the young population structure and overall short life expectancy in low-income countries [20]. The effect of genes, environment and lifestyle on this phenomenon is yet to be investigated. The average age at first childbirth is 20 years less than 29.4 years in Europe [21] and similar to the 19 years reported in lower income countries [22]. This was a couple with high parity, as almost a fifth of the patients had eight children or more. Parity is known as a protective factor against BC [23, 24]. Increased parity reduces the risk of BC but does not eliminate it; in fact, it may be associated with a higher risk of certain subtypes of cancer, like HER-2 enriched BC, as reported by Fortner *et al* [25]. Almost half of the patients were obese. A previous study in Eastern Sudan showed a higher prevalence of obesity compared to the Northern State and other African countries [26]. Obesity is associated with worse outcomes in BC [27, 28]. The high prevalence of obesity among patients with BC in Eastern Sudan necessitates the inclusion of lifestyle modification in management plans to improve outcomes [29].

More than 80% of participants presented at stage III or IV. The high percentage of females who present at late stages is slightly higher than the 60.7% and 65.3% reported in central Sudan [7, 30]. It is similar to the 74.7% of patients who are diagnosed at late stages in Sub-Saharan Africa [31] and contradictory to high-income countries where more than 70% of patients are diagnosed at stage I or II [32]. This late stage of presentation directly contributes to the high mortality of BC in low- and middle-income countries [33, 34]. BC screening via mammography is the most effective strategy to detect it early, but it can be economically challenging for low- and middle-income countries [35]. A recent systematic review showed that accurate clinical BC examination can be used as an alternative in low- and middle-income countries, but high-quality studies are needed to support it [35]. Almost half of the participants presented with grade 3 BC. A recent study demonstrated the prognostic value of grade, which can be used as an alternative to molecular methods in low- and middle-income countries [36]. In this study, grade 3 cancer was associated with an advanced stage of BC. Almost half of the patients had triple-negative BC. Triple-negative BC is common among black females, especially those who are younger than 40 years [37]. This is concerning given the aggressive nature and lack of targeted therapy for this cancer [38]. Interestingly, triple-negative BC was not significantly associated with advanced stages in our population.

The use of modified radical mastectomy is more common than breast conservative surgery for a large number of patients with advanced stage at presentation. Palliative treatment is needed by more than a third of patients, and more than a third of patients need radiotherapy. These patients are referred to other centres that offer radiotherapy in Wad Madani, Khartoum or Merowe, as radiotherapy is not available at EOC.

In this study, 20% of the patients had a family history of cancer, aligning with findings from a study of 6,706 women suggesting that familial or genetically predisposed BCs comprise approximately 15%–20% of cases [39]. Additionally, a family history of BC was negatively associated with the advanced stage of BC. Family history is a major risk factor for BC [40] and females with family history tend to present early, probably because they are more likely to be educated about BC [41]. We should build on this positive attitude and activate BC screening, especially for high-risk females. Future studies may focus on assessing these females for BRCA mutations and studying the effect of family history on ER, PR and HER2 status. Similarly, females with secondary school education tend to present at an earlier stage, emphasising the importance of education, which is highlighted in the literature [42–44].

This study described the clinicopathological characteristics of BC among patients receiving treatment at the EOC, which serves most of the population in Eastern Sudan. It adds to the knowledge of BC profiles in low- and middle-income countries like Sudan. The main limitation of this study is the limited and missing data caused by incomplete medical records in the centre. In the absence of a national cancer registry, the medical records of oncology centres carry extra value and should receive more care.

## Conclusion

The EOC covered 394 cancer patients in Eastern Sudan. Males constitute 4.3% of BC patients in the centre. Females tend to present at a young age and at an advanced stage. Almost half of the females were triple negative. A family history of BC was negatively associated with an advanced stage of BC. Grade 3 was positively associated with an advanced stage of BC.

## Conflicts of interest

The authors declare that they have no conflict of interest.

## Funding

The authors declare that this study was conducted without receiving any funding or financial support.

## Figures and Tables

**Table 1.** Demographic characteristics of the participants.

Characteristic	*N* = 394^a^
Gender	
Female	377 (96%)
Male	17 (4.3%)
Marital status	
Single	29 (7.4%)
Married	260 (66%)
Divorced	30 (7.6%)
Widowed	75 (19%)
Educational status	
Illiterate	117 (30%)
Informal	69 (18%)
Primary	96 (24%)
Secondary	76 (19%)
University	33 (8.4%)
Higher	3 (0.8%)
Occupation	
Cleaning worker	7 (1.8%)
Employee	10 (2.5%)
Farmer	8 (2.0%)
Free business	7 (1.8%)
Housewife	321 (81%)
Not working	5 (1.3%)
Others	10 (2.5%)
Teacher	26 (6.6%)
Residence	
Outside Gedaref state	79 (20%)
Rural area of Gedaref	168 (43%)
Urban area of Gedaref	147 (37%)

a *n* (%)

**Table 2.** BC-related characteristics of female patients.

Characteristic	*N* = 377^1^
Age at diagnosis	48 (38, 59)
Age at menarche^a^	14 (13, 15)
Age at menopause^a^	45 (41, 50)
Age at first birth^a^	20 (15, 25)
Parity^a^	
1–3	146 (42%)
4–7	134 (39%)
8 and more	64 (19%)
Family history of cancer	
No	302 (80%)
Yes	75 (20%)
BMI^a^	
Underweight	30 (12%)
Healthy weight	116 (45%)
Overweight	75 (29%)
Obese	35 (14%)
Stage	
I	6 (1.6%)
II	64 (17%)
III	188 (50%)
IV	119 (32%)
Grade^a^	
One	29 (8.1%)
Two	166 (46%)
Three	164 (46%)
Her2 positive^a^	
No	201 (75%)
Yes	67 (25%)
Her2 negative^a^	
No	72 (27%)
Yes	196 (73%)
Triple-negative breast cancer^a^	
No	153 (57%)
Yes	115 (43%)
Triple-positive breast cancer^a^	
No	117 (44%)
Yes	151 (56%)
Surgery	
Modified radical mastectomy	177 (47%)
Breast conservative surgery	81 (21%)
Radiotherapy	
No	232 (62%)
Yes	145 (38%)
Chemotherapy	
No	59 (16%)
Yes	318 (84%)
Hormonal therapy	
No	203 (54%)
Yes	174 (46%)
Palliative treatment	
No	228 (60%)
Yes	149 (40%)
Missed the follow-up	
No	374 (99%)
Yes	3 (0.8%)

^1a^Percentages may not sum to 100 because of missing data

Median (IQR); *n* (%)

**Table 3.** Predictors of advanced stage of BC patients in Eastern Sudan.

Characteristic	OR(95% CI)^1^	*p*-value
Marital status		
Single	—	
Married	1.66 (0.48–5.08)	0.39
Divorced	0.94 (0.21–4.07)	0.93
Widowed	4.30 (0.96–20.1)	0.056
Educational status		
Illiterate	—	
Informal	0.49 (0.16–1.51)	0.21
Primary	1.62 (0.51–5.32)	0.41
Secondary	0.28 (0.10–0.70)	**0.009**
University	0.33 (0.10–1.02)	0.053
Higher education	0.48 (0.04–11.7)	0.58
Family history of cancer		
No	—	
Yes	0.38 (0.18–0.78)	**0.009**
Breast cancer grade		
One	—	
Two	2.26 (0.82–6.11)	0.11
Three	3.78 (1.30–10.9)	**0.014**
Triple negative breast cancer		
No	—	
Yes	0.78 (0.39–1.54)	0.48
Her2 negative breast cancer		
No	—	
Yes	0.90 (0.09–7.83)	0.92
Her2 positive breast cancer		
No	—	
Yes	1.68 (0.15–16.1)	0.65

1 OR = Odds Ratio, CI = Confidence Interval

Adjusted R squared:0.14

Hosmer-Lemeshow goodness of fit (GOF) test:

*X*-squared = 2.0399, degree of freedom = 8, *p*-value = 0.9798
